# Effects of Phase-Locking Deficits on Speech Recognition in Older Adults With Presbycusis

**DOI:** 10.3389/fnagi.2018.00397

**Published:** 2018-12-06

**Authors:** Wenyang Hao, Qian Wang, Liang Li, Yufei Qiao, Zhiqiang Gao, Daofeng Ni, Yingying Shang

**Affiliations:** ^1^Department of Otorhinolaryngology, Peking Union Medical College Hospital, Chinese Academy of Medical Sciences and Peking Union Medical College, Beijing, China; ^2^Epilepsy Center, Department of Clinical Psychology, Sanbo Brain Hospital, Capital Medical University, Beijing, China; ^3^School of Psychological and Cognitive Sciences and Beijing Key Laboratory of Behavior and Mental Health, Speech and Hearing Research Center, Key Laboratory on Machine Perception (Ministry of Education), Peking University, Beijing, China

**Keywords:** frequency following response, presbycusis, auditory aging, auditory brainstem response, speech recognition

## Abstract

**Objective**: People with presbycusis (PC) often report difficulties in speech recognition, especially under noisy listening conditions. Investigating the PC-related changes in central representations of envelope signals and temporal fine structure (TFS) signals of speech sounds is critical for understanding the mechanism underlying the PC-related deficit in speech recognition. Frequency-following responses (FFRs) to speech stimulation can be used to examine the subcortical encoding of both envelope and TFS speech signals. This study compared FFRs to speech signals between listeners with PC and those with clinically normal hearing (NH) under either quiet or noise-masking conditions.

**Methods**: FFRs to a 170-ms speech syllable /da/ were recorded under either a quiet or noise-masking (with a signal-to-noise ratio (SNR) of 8 dB) condition in 14 older adults with PC and 13 age-matched adults with NH. The envelope (FFR_ENV_) and TFS (FFR_TFS_) components of FFRs were analyzed separately by adding and subtracting the alternative polarity responses, respectively. Speech recognition in noise was evaluated in each participant.

**Results**: In the quiet condition, compared with the NH group, the PC group exhibited smaller F0 and H3 amplitudes and decreased stimulus-response (S-R) correlation for FFR_ENV_ but not for FFR_TFS_. Both the H2 and H3 amplitudes and the S-R correlation of FFR_ENV_ significantly decreased in the noise condition compared with the quiet condition in the NH group but not in the PC group. Moreover, the degree of hearing loss was correlated with noise-induced changes in FFR_TFS_ morphology. Furthermore, the speech-in-noise (SIN) threshold was negatively correlated with the noise-induced change in H2 (for FFR_ENV_) and the S-R correlation for FFR_ENV_ in the quiet condition.

**Conclusion**: Audibility affects the subcortical encoding of both envelope and TFS in PC patients. The impaired ability to adjust the balance between the envelope and TFS in the noise condition may be part of the mechanism underlying PC-related deficits in speech recognition in noise. FFRs can predict SIN perception performance.

## Introduction

Presbycusis (PC) is the third most common chronic disorder in elderly people, reflecting the degradation of auditory-processing functions in both peripheral and central systems (Yueh et al., 2003). Listeners with PC often manifest both symmetrical sensorineural hearing loss (SNHL) and impaired speech recognition (Deng et al., 2014), especially in noisy environments (Li et al., 2004; Divenyi et al., 2005; Gifford et al., 2007; Huang et al., 2008; Salonen et al., 2013). However, the link between PC and augmented vulnerability of speech recognition to noise masking is still not completely understood.

After a soundwave (such as speech sound) reaches the ears, the peripheral auditory system filters the sound wave into bands of narrowband waves through a series of band-pass filters, and the output signals from each of the narrowband channels are further decomposed into fast fluctuating temporal fine structures (TFSs) and slowly varying envelopes (ENVs; Moore, 2008). Considerable evidence has suggested that auditory aging markedly affects the detection of both the TFS and envelope components (Füllgrabe et al., 2003, 2015; Buss et al., 2004; Lorenzi et al., 2006, 2012; Souza and Boike, 2006; Hopkins and Moore, 2007; Fogerty and Humes, 2012; Moore et al., 2012; Füllgrabe, 2013; Rufener et al., 2016). It is of interest and importance to determine whether the age-related deficits in processing TFS and envelope signals start to occur at the level of the auditory brainstem.

Scalp-recorded frequency-following responses (FFRs) are sustained neuro-electrical potentials representing the periodicity of acoustic stimuli (Worden and Marsh, 1968; Moushegian et al., 1973) with the origin site in the auditory midbrain, including the inferior colliculus (Weinberger et al., 1970; Marsh et al., 1974; Smith et al., 1975; Sohmer et al., 1977; Ping et al., 2008; Du et al., 2009; Chandrasekaran and Kraus, 2010; Bidelman, 2015; Wang and Li, 2015, 2018; Luo et al., 2017). Both the sound TFS component (e.g., Galbraith, 1994; Krishnan, 2002; Krishnan and Gandour, 2009; Chandrasekaran and Kraus, 2010; Du et al., 2011) and the envelope component (also called the envelope-following response or the steady-state evoked response; e.g., Hall, 1979; Dolphin and Mountain, 1992, 1993; Supin and Popov, 1995; Russo et al., 2004; Aiken and Picton, 2006, 2008; Shinn-Cunningham et al., 2013; Zhu et al., 2013) are represented in the FFRs and are, therefore, useful for studying the mechanisms underlying speech recognition in noisy environments (Du et al., 2011). In both humans and rats, auditory brainstem responses (ABRs) induced by complex sound signals (e.g., speech syllables composed of consonants and vowels) or noises contain both transient responses and sustained FFRs (Skoe and Kraus, 2010; Wang and Li, 2018), and both the TFS component (FFR_TFS_) and the envelope component (FFR_ENV_) of FFRs can be assessed independently (Aiken and Picton, 2008).

To date, several studies have used FFR recordings to investigate how aging affects auditory processing. For instance, compared with younger adults with normal hearing (NH), older adults with clinically NH exhibit weakened phase locking and reduced FFR magnitudes (Anderson et al., 2012; Clinard and Tremblay, 2013; Bidelman et al., 2014). Additionally, among older adults with NH, the fundamental frequency (F0) magnitude of FFRs is larger and less affected by noise in those who perform better on the speech-in-noise (SIN) test (Anderson et al., 2011). Thus, older adults lose temporal precision in the subcortical encoding of sounds, leading to difficulty with speech perception against masking. It is important to determine whether the aging effects are enhanced by PC and particularly whether the extent of age-related brain atrophy is associated with central hearing loss in older adults (Giroud et al., 2018).

Recently, Ananthakrishnan et al. (2016) examined FFRs in adult listeners with or without hearing loss and revealed that the neural representations of the envelopes and TFSs are weaker in listeners with SNHL. However, Ananthakrishnan et al.’s included young and middle-aged NH listeners and SNHL patients with a wide range of ages rather than only listeners with PC. Thus, further studies are needed to clarify how the aging effects are modulated by audibility.

In older adults with or without hearing loss, Anderson et al. (2013a) reported greater auditory-nerve coding of sound envelopes and no significant difference in the TFS in the SNHL groups. The stimulus used in their study was a short stimulus (e.g., 40 ms /da/), which did not have a real steady-state vowel of the syllable, although it could be perceived as a consonant-vowel syllable. Thus, their results may not be able to accurately represent the subcortical encoding ability of the envelope and the TFS. Therefore, in listeners with PC, the exact effect of audibility deficits on the subcortical encoding of the envelope and the TFS remains unclear. Furthermore, although the FFR in noise conditions is more likely to reflect speech recognition in noise than the FFR in quiet conditions, Ananthakrishnan et al. (2016) did not test the FFR in noise conditions. The study by Anderson et al. (2013a) was the only to examine FFR in noise, and data on the FFR in listeners with PC are still very scarce. In addition, it has also been demonstrated that experience with tonal languages can affect neural plasticity at the brainstem level (Krishnan and Gandour, 2009; Krishnan et al., 2010a,b). However, most previous FFR studies included participants who were not native speakers of a tonal language. Thus, systematic studies using FFR to examine how envelope and TFS detection is affected by audibility deficits and how noise can affect envelope and TFS subcortical encoding in listeners with PC, especially native speakers of a tonal language, could be informative.

In the present study, we examined FFR under both quiet and noise conditions in older adults with and without PC who were native speakers of a tonal language (Mandarin) and analyzed the FFR results together with their pure tone audiometry (PTA) thresholds and SIN performance. We hypothesized that the loss of hearing sensitivity and the reduction in SIN perception of listeners with PC, particularly when listening under noise conditions, may be associated with the decline in the subcortical representations of the envelope and TFS signals, which can be measured in both humans (Wang et al., 2018) and laboratory animals (Wang and Li, 2015, 2017, 2018; Luo et al., 2017). The primary aim of the present study was to obtain a better understanding of the PC-related alterations in the neural representation of envelope and TFS cues.

## Materials and Methods

### Participants

Fourteen older adults (≥60 years old) with PC and 13 age-matched older adults with clinically NH participated in the present study. NH was defined as the following: (1) PTA air conduction thresholds no higher than 25 dB HL from 500 Hz to 3,000 Hz bilaterally (Pross et al., 2015); (2) air-bone gaps no larger than 10 dB HL; and (3) no interaural asymmetry (a difference no larger than 15 dB HL at two or more frequencies). The aged adults with PC included only those with mild to moderate symmetric SNHL, defined as follows: (1) air-bone gaps ≤10 dB HL; (2) air conduction thresholds higher than 25 dB HL for the frequencies from 500 Hz to 3,000 Hz; and (3) no interaural asymmetry (≤15 dB HL difference at two or more frequencies). All participants underwent tympanometry, and those with abnormal results were excluded. Additionally, all participants had normal cognitive abilities, as measured with the Mini-Mental State Examination (MMSE, ≥27). All participants were right handed. No participants reported any history of hearing aid usage. No participants reported any history of neurological conditions. Those who reported a history of musical training (>3 years) were also excluded.

PTA was performed using a Conera audiometer (Madsen, GN, Denmark; ISO 389). Frequencies from 0.25 kHz to 8 kHz were tested using headphones TDH 39 with a step size of 5 dB HL (ISO 8253-1:1989). The SIN test was assessed using the Hearing-in-Noise Test (HINT; Bio-logic Systems Corp., Mundelein, IL, USA). Ten-word sentences from 12 different Mandarin lists consisting of 20 sentences each were presented randomly during the SIN threshold tests. Mandarin HINT sentences were presented through the headphones at different intensities with an ipsilateral fixed speech-shaped noise masker (65 dB sound pressure level, dB SPL). The sentence was presented beginning at a −10 dB signal-to-noise ratio (SNR) and adapted to be easier or more difficult based on each participant’s responses. The step size was 4 dB for the first four sentences and 2 dB for the remaining 16 sentences. The SIN threshold was the average of the presenting SNR from sentence No. 5 through 20 (Nilsson et al., 1994).

This study was carried out in accordance with the recommendations of Declaration of Helsinki-Ethical Principles for Medical Research Involving Human Subjects, World Medical Association. The procedures used in this study were approved by the Ethics Committee of Peking Union Medical College Hospital, and all participants provided their written informed consent.

### FFR Recordings

**Figure 1:**
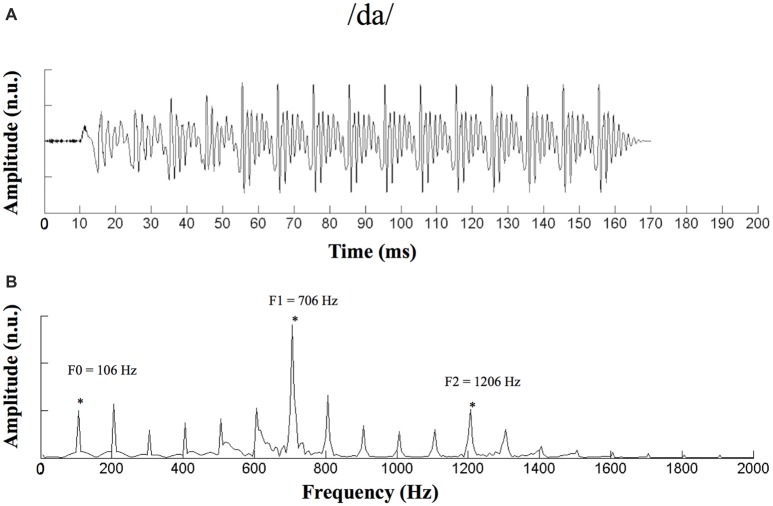
Waveform and spectrogram of the stimulus /da/. **(A)** Waveform of the 170-ms /da/ stimulus. **(B)** Spectrogram of the /da/ stimulus. n.u., no unit. *Represent the peak of F0 and its harmonics.

A 170-ms speech syllable, /da/ (Anderson et al., 2011, 2012; Mamo et al., 2016), which is an important elemental speech cue in Mandarin, was used as the stimulus (Figure 1). This syllable consisted of a 50-ms transition (from the stop burst of [d] to [a]) followed by a 120-ms steady-state region corresponding to the vowel [a]. During the steady-state region, the fundamental frequency (F0) remained constant at 100 Hz, and the first and second formant-related harmonics remained at 720 Hz (F1) and 1240 Hz (F2), respectively (Anderson et al., 2011, 2012).

An auditory evoked-potential-recording system (SmartEP, Intelligent Hearing Systems (IHS), Miami, FL, USA) was used to record ABRs to the speech stimulus. The /da/ stimulus was presented monaurally through electromagnetically shielded insert earphones (ER-3A) at an intensity of 85 dB SPL and a rate of 3.89 Hz (Anderson et al., 2013a; Ananthakrishnan et al., 2016). ABRs were recorded under both the quiet and noise conditions. For the noise condition, continuous white noise was presented ipsilaterally at an SNR of 8 dB. A vertical montage of four silver disc electrodes (Cz active, Fpz ground, mastoid references) was used with interelectrode impedances maintained below 5 kΩ for all recordings. The sampling rate was 2.5 kHz, and the band-pass filter was from 30 Hz to 3,000 Hz. Under either the quiet or noise condition for each ear, a block of 2,048 sweeps was collected separately for the condensation and rarefaction polarities and averaged using a 240-ms window (−40 to 200 ms). To ensure that the participants remained awake and relaxed, they were instructed to watch a muted, subtitled movie of their choice while sitting on a couch. ABR recordings were made in an electrically shielded, sound-proof booth.

### Data Analyses

To extract the noninverting FFR_ENV_, responses to the two different polarities were added, while responses to the two different polarities were subtracted to extract the inverted FFR_TFS_ (Aiken and Picton, 2008). Spectral amplitudes were computed using fast Fourier transformations for both FFR_ENV_ and FFR_TFS_ to decompose their component frequencies on a time window of 60–170 ms, which corresponded to the steady-state region of the responses. The bin size was 4.88 Hz.

For the spectral analysis of FFR_ENV_, the amplitude of F0 (100 Hz) and the second and third harmonics (H2 and H3, 200 Hz and 300 Hz, respectively) were analyzed. For FFR_TFS_, the amplitude of F1 (720 Hz) was analyzed. Stimulation-response (S-R) correlations were assessed for both FFR_ENV_ and FFR_TFS_ of each ear and each condition by calculating Pearson’s *r* value between the response and the /da/ stimulus from 0 ms to 170 ms. The FFR_TFS_ and FFR_ENV_ components were also separated by adding and subtracting responses to the two different polarities described above. The value with the optimal delay (which was associated with the maximum S-R correlation) was used to assess the S-R correlation coefficient. Cross-correlations were also used to evaluate the similarity between the responses in the quiet and noise conditions (Anderson et al., 2011). To evaluate the changes in waveform morphology induced by noise, correlation coefficients were calculated by shifting the response waveform obtained in the noise condition relative to the response waveform obtained in the quiet condition (±2 ms). The maximum correlation achieved (in terms of Pearson’s *r* value) was defined as the quiet-to-noise response correlation value. Fisher’s transformation was used to convert the *r* values to *z* scores for statistical analyses.

Statistical analyses were performed using SPSS 16.0 (SPSS, Inc., Chicago, IL, USA). Analysis of variance (ANOVA) was used for group (NH, PC) comparisons of the F0 amplitude and its multiple harmonic peaks. Independent sample *t*-tests were used to determine the differences in age, pure tone thresholds, SIN thresholds, S-R correlations and quiet-to-noise responses between the groups. Paired *t*-tests were used to compare the amplitude alterations of F0 and its multiple harmonic peaks between quiet and noise conditions. Paired *t*-tests were also used to compare S-R correlations between quiet and noise conditions. To explore the continuous relationships among age, PTA threshold, SIN threshold and FFR variables, Pearson’s correlation was used. The Bonferroni correction was used for multiple comparisons.

## Results

### Demographic and Audiology Results

**Figure 2:**
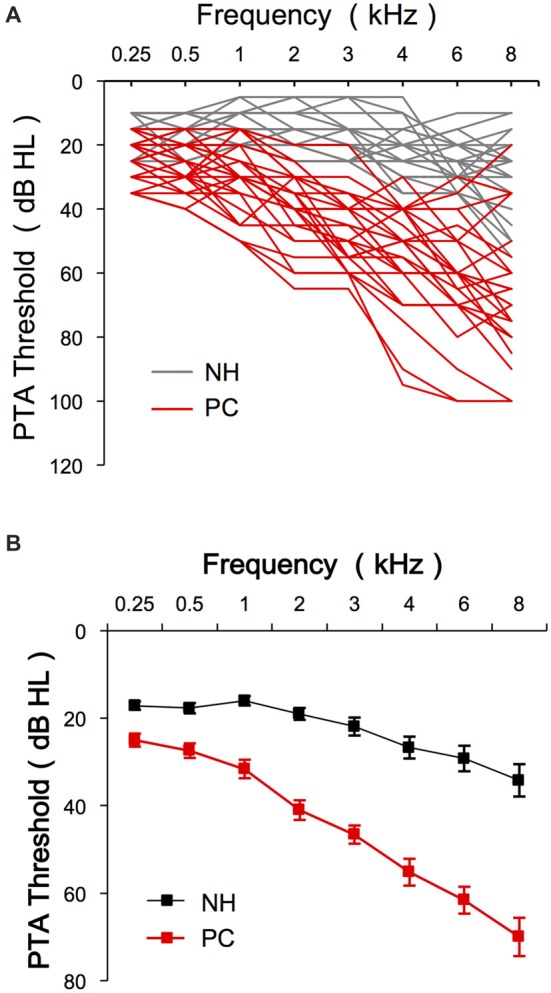
Audiology data of the participants. **(A)** PTA thresholds of each subject for both NH and PC groups. **(B)** Average PTA thresholds for the NH group and the PC group. Error bars represent SEM. NH, normal hearing; PC, presbycusis; PTA, pure tone audiometry.

The present study enrolled 13 older adults with NH (M/F = 6/7, age 60–74 years, average 63.1) and 14 older adults with PC (M/F = 10/4, age 60–82 years, average 65.9; Table 1). No significant age difference was found between the two groups (*t* = −1.339, *p* = 0.192). The PTA thresholds for both groups are shown in Figure 2. The SIN threshold results are also presented in Table 1, and a *t*-test revealed significantly higher SIN thresholds in the PC group than in the NH group (*t* = 7.274, *p* < 0.001).

**Table 1:** Demographic and audiology results.

	NH (*n* = 13)	PC (*n* = 14)
Sex (Male/Female)	6/7	10/4
Age (Mean/SD)	63.1/4.4	65.9/6.3
PTA threshold (Mean/SD)	19.9/4.8	42.5/8.0
SIN threshold (Mean/SD)	2.0/3.7	4.0/1.9

*NH, normal hearing; PC, presbycusis; PTA, pure tone audiometry; SIN, speech-in-noise*.

### Amplitude of F0 and Its Harmonics

**Figure 3:**
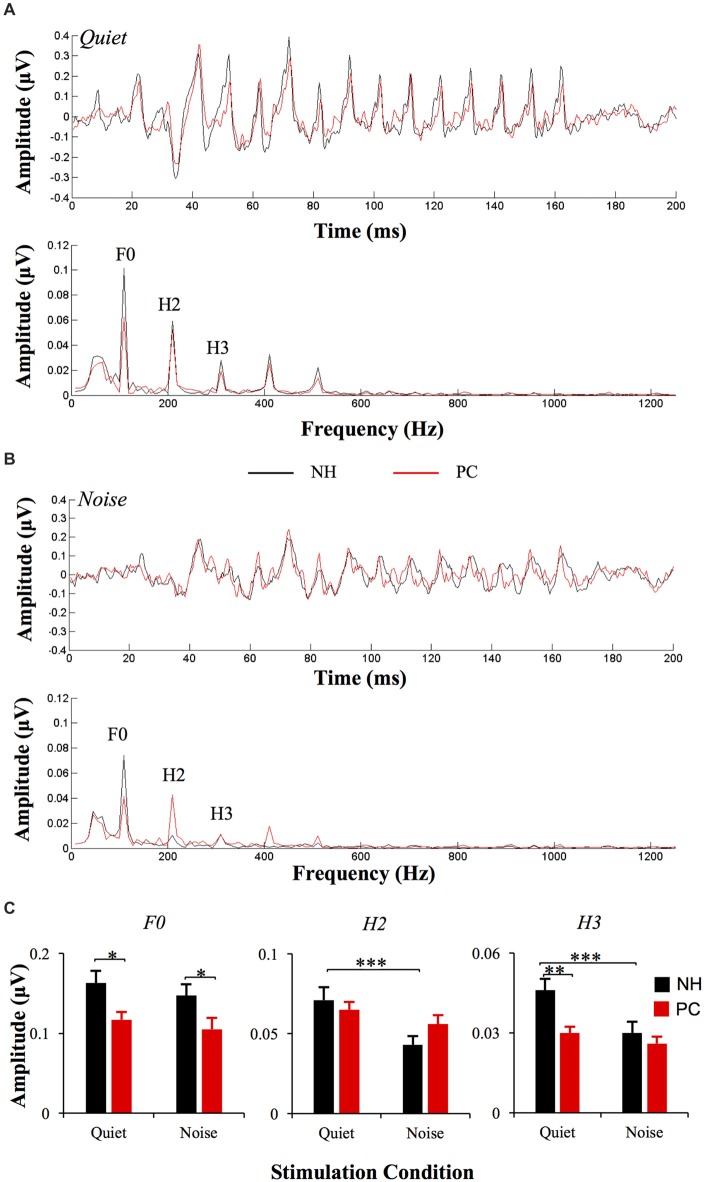
Comparison of FFR_ENV_ between the NH and PC groups. **(A)** Grand average waveforms and spectrograms of FFR_ENV_ for the NH and PC groups under the quiet condition. **(B)** Grand average waveforms and spectrograms of FFR_ENV_ for the NH and PC groups under the noise condition. **(C)** Amplitude comparison of F0, H1 and H2 (FFR_ENV_) between the NH and PC groups under both quiet and noise conditions. NH, normal hearing; PC, presbycusis; FFR, frequency-following response; ENV, envelope. *p < 0.05, **p < 0.01; ***p < 0.05/8 = 0.006, Bonferroni corrected.

The responses of two different polarities were added for each condition of each participant to extract the FFR_ENV_. The average response waveforms of FFR_ENV_ for each group and their spectrograms are presented in Figure 3. According to the average waveforms, the PC group had lower FFR responses than the NH group in the quiet condition; however, in the noise condition, there was no significant amplitude difference between the NH and PC groups. According to the spectrogram of the grand average response in the quiet condition, the amplitudes of F0 and its harmonics for the PC group were lower than those for the NH group. However, in the noise condition, all the harmonics of the PC group showed higher amplitudes than those of the NH group except for F0, which was much lower in the PC group.

**Figure 4:**
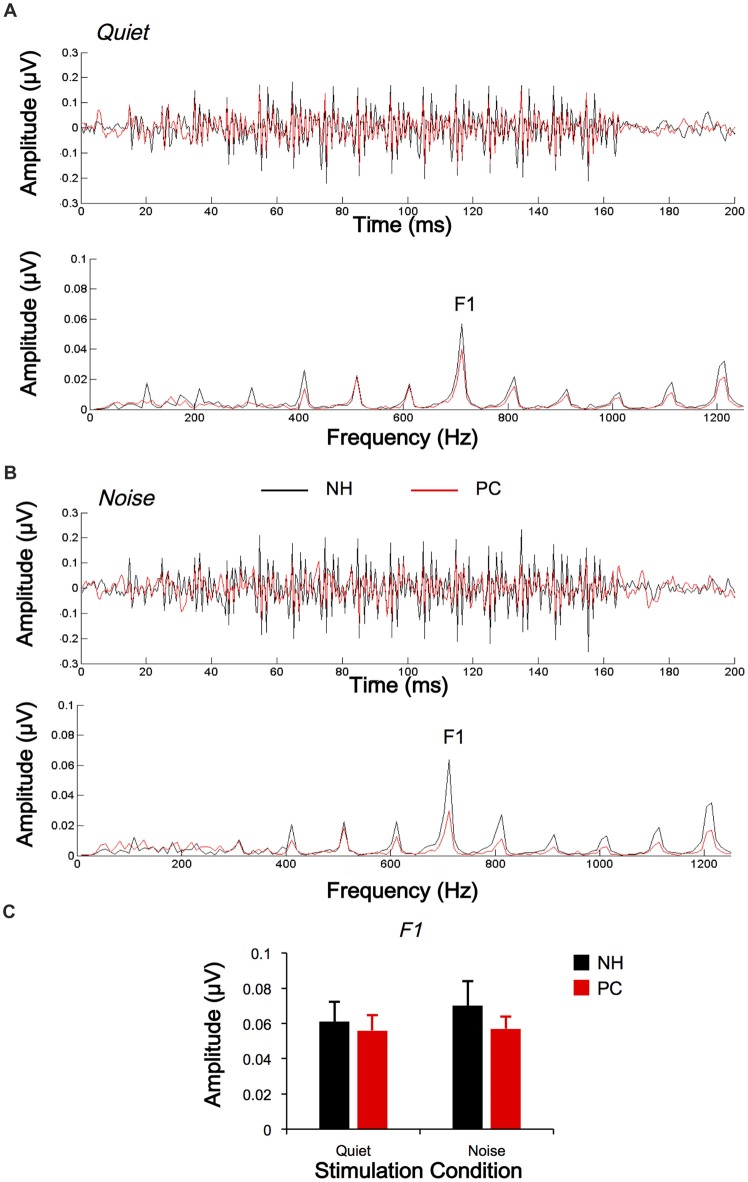
Comparison of FFR_TFS_ between the NH and PC groups. **(A)** Grand average waveforms and spectrograms of FFR_TFS_ for the NH and PC groups under the quiet condition. **(B)** Grand average waveforms and spectrograms of FFR_TFS_ for the NH and PC groups under the noise condition. **(C)** Amplitude comparison of F1 (FFR_TFS_) between the NH and PC groups under both quiet and noise conditions. NH, normal hearing; PC, presbycusis; FFR, frequency-following response; TFS, temporal fine structure.

The responses of two different polarities were also subtracted for each condition and each participant to extract the FFR_TFS_. The average response waveforms of FFR_TFS_ for each group and their spectrograms are shown in Figure 4. According to the average response waveforms, the FFR_TFS_ of the PC group showed a lower amplitude than that of the NH group under both the quiet and noise conditions, but there were no obvious amplitude differences between the two conditions for either group. According to the spectrograms, the amplitude of F1 for the NH group was higher in the noise condition than in the quiet condition, while no similar phenomenon was observed for the PC group.

We also quantitatively compared the amplitudes of F0 and its harmonics between the two participant groups using multivariate ANOVA (Table 2, Figures 3C, 4C), and the trends were similar to those of the average waveform and spectrogram. The overall intergroup effect was significant (*F*_(1,52)_ = 4.124, *p* = 0.001). The amplitudes of F0 and H3 in the quiet condition and F0 in the noise condition were significantly lower in the PC group than in the NH group, and no significant difference was detected for FFR_TFS_ between groups. We also investigated the effect of noise on the amplitudes of F0 and its harmonics by comparing the amplitudes between quiet and noise conditions using paired *t*-tests (Table 2, Figures 3C, 4C). Compared with the quiet condition, H2 and H3 showed significantly decreased amplitudes (*p* < 0.001; *p* < 0.05/8 = 0.006, Bonferroni corrected), while no significant difference was observed for the PC group or for TFS. Furthermore, group differences in the amplitude change caused by noise were also compared, and the PC group showed significantly smaller changes for H2 and H3.

**Table 2:** FFR comparison between aged people with and without hearing loss.

	Quiet			Noise			Quiet vs. Noise		Quiet-Noise		
	NH	PC		NH	PC		NH	PC	NH	PC	
	Mean (SD)	Mean (SD)	*F*(*p* value)	Mean (SD)	Mean (SD)	*F* (*p* value)	*t* (*p* value)	*t* (*p* value)	Mean (SD)	Mean (SD)	*F* (*p* value)
ENV											
F_0_	0.163 (0.077)	0.117 (0.051)	6.518 (0.014)*	0.147 (0.073)	0.105 (0.060)	5.098 (0.028)*	1.305 (0.204)	1.590 (0.124)	0.016 (0.061)	0.012 (0.039)	0.086 (0.770)
H_2_	0.071 (0.041)	0.065 (0.026)	0.490 (0.487)	0.043 (0.028)	0.056 (0.030)	2.882 (0.096)	4.129 (0.000)***	1.792 (0.084)	0.028 (0.035)	0.008 (0.024)	5.971 (0.018)*
H_3_	0.046 (0.022)	0.030 (0.012)	12.004 (0.001)**	0.030 (0.021)	0.026 (0.014)	0.847 (0.362)	4.063 (0.000)***	1.427 (0.165)	0.016 (0.020)	0.004 (0.015)	6.126 (0.017)*
TFS											
F_1_	0.061 (0.058)	0.056 (0.046)	0.091 (0.765)	0.070 (0.071)	0.057 (0.037)	0.731 (0.396)	−2.087 (0.049)	−1.639 (0.114)	−0.009 (0.022)	−0.000 (0.035)	1.178 (0.283)

*p < 0.05; **p < 0.01; ***p < 0.05/8 = 0.006, Bonferroni corrected. NH, normal hearing; PC, presbycusis; FFR, frequency-following response; ENV, envelope; TFS, temporal fine structure. Bold values represent p < 0.05.

In addition, in order to evaluate the group and condition effects, 2 (group: NH, PC) × 2 (condition: quiet, noise) two-way mixed-measured ANOVAs were conducted to examine the effects on the F0 amplitude and its multiple harmonic peaks, respectively. ANOVAs showed that the main effects of condition were significant for H2 (*F*_(1,52)_ = 20.067, *p* < 0.001) and H3 (*F*_(1,52)_ = 17.388, *p* < 0.001), but not for either F0 (*F*_(1,52)_ = 3.891, *p* = 0.054) or F1 (*F*_(1,52)_ = 1.242, *p* = 0.270). The main effects of group were significant for F0 (*F*_(1,52)_ = 6.801, *p* = 0.012) and H3 (*F*_(1,52)_ = 6.465, *p* = 0.014), but not for either H2 (*F*_(1,52)_ = 0.201, *p* = 0.656) or F1 (*F*_(1,52)_ = 0.373, *p* = 0.544). The interaction effect was significant for H2 (*F*_(1,52)_ = 5.971, *p* = 0.018) and H3 (*F*_(1,52)_ = 6.126, *p* = 0.017), but not for either F0 (*F*_(1,52)_ = 0.086, *p* = 0.770) or F1 (*F*_(1,52)_ = 1.178, *p* = 0.283).

### Stimulus-Response Correlation

**Figure 5:**
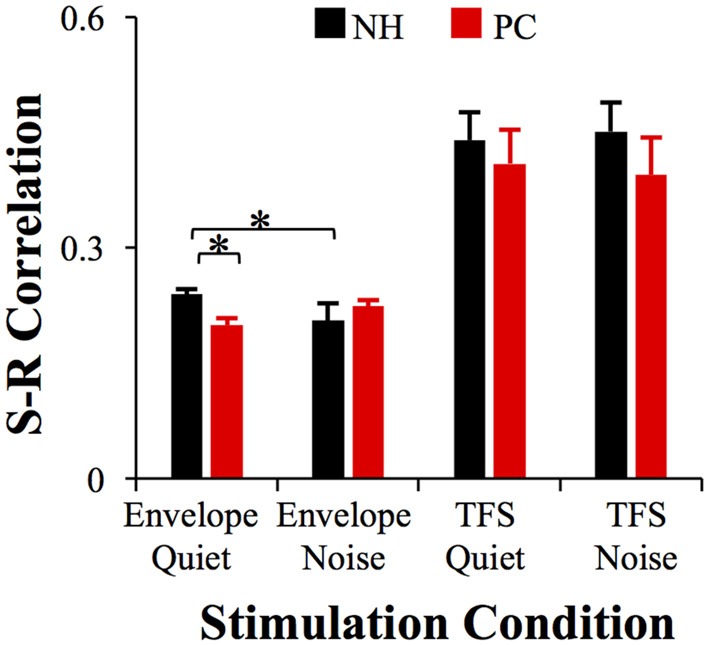
Comparison of the S-R correlation between the NH and PC groups. The S-R correlation of FFR_ENV_ in the NH group in the quiet condition was significantly higher than that of the PC group in the quiet condition, and was also higher than that of the NH group in the noise condition. No significant differences were observed in FFR_TFS_ between groups or conditions. NH, normal hearing; PC, presbycusis; FFR, frequency-following response; ENV, envelope; TFS, temporal fine structure; S-R, stimulus-response. *p < 0.05/4 = 0.01, Bonferroni corrected.

S-R correlations were analyzed for both FFR_ENV_ and FFR_TFS_ to reflect the accuracy of subcortical phase-locking encoding. Pearson’s correlation tests showed that the S-R correlation between the FFRENV and the acoustic /da/ was significant (all *p* < 0.05); the S-R correlation between the FFRTFS and the acoustic /da/ was also significant (*p* < 0.05) except for one tested ear in the quiet condition. For the NH group, the S-R correlation of FFR_ENV_ in the quiet condition was significantly higher than that in the noise condition (*t* = 3.735, *p* = 0.001; *p* < 0.05/4 = 0.01, Bonferroni corrected), while no such difference was observed for the PC group (Figure 5). In the quiet condition, the NH showed a significantly higher S-R correlation of FFR_ENV_ than the PC group (*t* = 3.487, *p* = 0.001; *p* < 0.05/4 = 0.01, Bonferroni corrected), while no such difference was observed in the noise condition (Figure 5). No significant differences were observed for FFR_TFS_ between groups or conditions.

### FFR Morphology Affected by Noise

**Figure 6:**
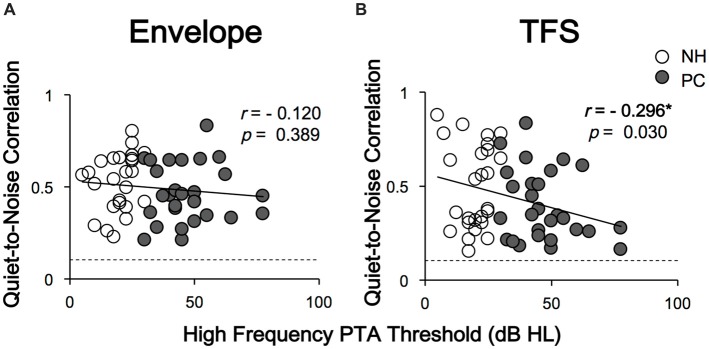
Correlations between high-frequency PTA thresholds and quiet-to-noise correlations. Panel **(A)** shows that the quiet-to-noise correlation of FFR_ENV_ was not significantly correlated with the high-frequency PTA threshold. Panel **(B)** shows that the quiet-to-noise correlation of FFR_TFS_ was negatively correlated with the high-frequency PTA threshold. The dash lines indicate the *r* values corresponding to the *p* value of 0.05. The *r* values higher than the dash lines indicate significant correlations between responses in quiet and those under the noise conditions, i.e., *p* < 0.05. Except for FFR_TFS_ of one tested ear in quiet condition, all response-stimulus correlations were significant. NH, normal hearing; PC, presbycusis; FFR, frequency-following response; TFS, temporal fine structure. *p < 0.05.

To evaluate the influence of noise on FFR morphology, Pearson’s correlation between the responses of the two conditions (quiet and noise) was calculated, and a significant correlation was found between all tested ears (all *p* < 0.05). Correlations between age, PTA thresholds, SIN thresholds and the *r* values of quiet-to-noise response correlations were also evaluated using Pearson’s correlation analysis. The results indicated a negative relationship between high-frequency PTA thresholds (2 and 4 kHz) and *r* values for FFR_TFS_ (*r* = −0.296, *p* = 0.030, Figure 6), in which higher high-frequency PTA thresholds were associated with an increased impact of noise on response morphology. No significant relationship was revealed for age. No* r* value differences between the NH and PC groups were detected for either FFR_ENV_ or FFR_TFS_.

### Correlation Between FFR and SIN Recognition

**Figure 7:**
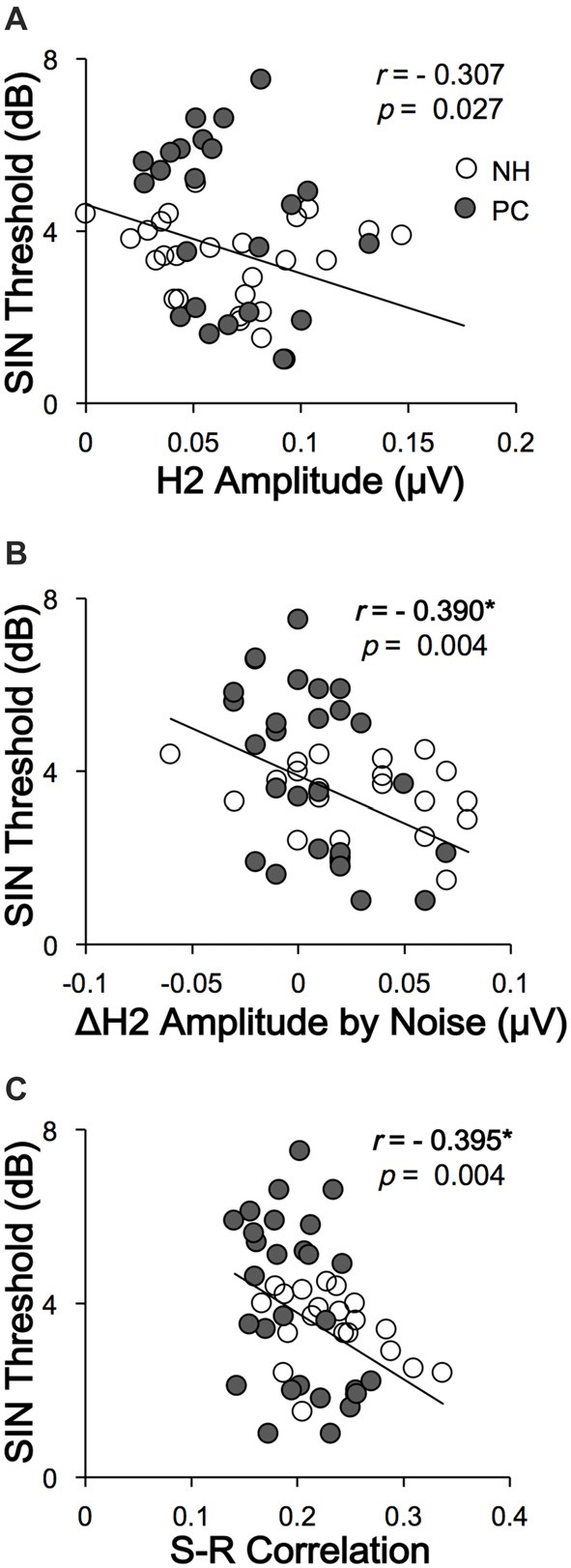
Correlations between SIN recognition performances and FFR variables. **(A)** The SIN recognition thresholds were not significantly correlated with H2 (FFR_ENV_) amplitude in the quiet condition. **(B)** Higher SIN recognition thresholds (worse performance) were significantly correlated with lower H2 (FFR_ENV_) amplitude alterations in the noise condition. **(C)** Higher SIN recognition thresholds (worse performance) were correlated with lower FFR_ENV_ S-R correlations in the quiet condition. NH, normal hearing; PC, presbycusis; FFR, frequency-following response; ENV, envelope; SIN, speech-in-noise; S-R, stimulus-response. *p < 0.05/11 = 0.005, Bonferroni corrected.

To clarify whether FFRs can predict SIN perception performance, the correlations between SIN thresholds and FFR variables were also assessed. The results showed that higher SIN thresholds (worse SIN performance) were significantly related to lower H2 (FFR_ENV_) amplitude alteration induced by noise (*r* = −0.390, *p* = 0.004; *p* < 0.05/11 = 0.005, Bonferroni corrected) and lower FFR_ENV_ S-R correlation under quiet conditions (*r* = −0.395, *p* = 0.004; *p* < 0.05/11 = 0.005, Bonferroni corrected; Figure 7). No significant correlation was found for the quiet-to-noise correlation, amplitude of F0 or its harmonics, or FFR_TFS_ S-R correlation.

## Discussion

This study investigated the FFR in elderly adults with or without PC under quiet and noise conditions. The results showed that the elevated hearing sensitivity in listeners with PC affected subcortical encoding of both the envelope and TFS. The main findings are as follows: (1) under quiet conditions, the F0 and H3 amplitudes and the S-R correlation of FFR_ENV_ in the PC group were significantly lower than those in the NH group, but the F1 amplitudes and the S-R correlation of the FFR_TFS_ exhibited no significant differences; (2) under noise conditions, the H2 and H3 amplitudes and S-R correlation of FFR_ENV_ in the NH group significantly decreased compared with those under quiet conditions, but no similar alteration was observed in the PC group or for FFR_TFS_; (3) the higher degree of hearing loss was correlated with greater changes in TFS morphology caused by noise; and (4) better SIN performance was closely related to higher FFR_ENV_ S-R correlation in the quiet condition and higher H2 (FFR_ENV_) amplitude alteration in the noise condition.

### Influence of Audibility on the Neural Representation of the Envelope and TFS in the Quiet Condition

In this study, the results of FFR testing under quiet conditions showed that for FFR_ENV_, the F0 and H3 amplitudes of the PC group were significantly lower than those of the NH group, suggesting that patients with reduced audibility had a decreased subcortical ability to encode the envelope. The PC group also exhibited lower response-stimulus correlations of FFR_ENV_ than the NH group, which suggested that hearing loss led to less accuracy of subcortical encoding for envelope cues. These findings suggested that the ability of subcortical encoding of the envelope decreased with hearing loss. Ananthakrishnan et al. (2016) also found that SNHL patients had a lower F0 amplitude of FFR_ENV_ than individuals with NH in response to stimuli of the same intensity, a finding that is consistent with the results of this study. However, Anderson et al. (2013a) showed that the F0, H1 and H2 amplitudes of the FFR_ENV_ of patients with hearing loss were higher than those of individuals with NH. This discrepancy is likely derived from the different hearing sensitivities of the subjects included in the studies. In the study by Anderson et al. (2013a), the average hearing threshold of the subjects with hearing loss was significantly lower, indicating better hearing, than that of the hearing-impaired subjects in this study and the study by Ananthakrishnan et al. (2016).

For TFS, the sum waveforms and their spectra showed that the F1 amplitudes of the PC group were lower than those of the NH group. These findings still suggest that under quiet conditions, hearing sensitivity exerts a significant influence on subcortical TFS encoding ability, although the multivariate ANOVA did not show significant amplitude or S-R correlation differences between the two groups. Anderson et al. (2013a,c) and Ananthakrishnan et al. (2016) also investigated TFS in SNHL patients. In one of their previous studies, Anderson et al. (2013a) did not find significant differences in subcortical encodings of TFS between the NH group and the PC group. However, another study implementing a larger group of participants revealed TFS deficits in the PC group (Anderson et al., 2013c), which is consistent with Ananthakrishnan et al.’s (2016) reports. Thus, the lack of statistically significant differences in our study was probably due to the relatively small sample size.

### Influence of audibility on the Neural Representation of the Envelope and TFS in the Noise Condition

In this study, we also investigated the FFR under noise conditions. The results showed that in the NH group, the amplitudes of H2 and H3 (FFR_ENV_) in the noise condition were significantly decreased compared with in the quiet condition, and the S-R correlation also decreased. However, no significant decrease was observed for TFS. These findings suggest that in noise conditions, the proportion of information extracted by the subcortical nuclei had changed, and the TFS proportion appeared to have increased compared with quiet conditions; in contrast, the envelope proportion decreased. Therefore, during the speech recognition process, subjects with NH were more likely to depend on TFS-related information under noise conditions than under quiet conditions. Bidelman (2016) investigated FFR using processed speech stimuli containing only ENV or TFS cues, and the neuro-acoustic and response-to-response correlations revealed that speech-FFRs were dominated by the stimulus ENV for clean speech, with TFS making a stronger contribution at moderate noise levels. Our results further supported their findings.

However, in the PC group, no significant amplitude or S-R correlation difference for FFR_ENV_ was found between the two conditions. Moreover, the scale of the amplitude change of H2 and H3 under the two conditions in the PC group was lower than that in the NH group, suggesting that the energy change of FFR_ENV_ in the PC group was significantly lower than that in the NH group. These findings suggest that the PC subjects were not able to downgrade the envelope information extraction in the noise condition like the NH subjects. The results of this study are consistent with a previous study investigating the neural encoding of the ENV in SNHL animals (Zhong et al., 2014), which showed that hearing loss is not associated with a stronger adverse effect of increasing masker intensity on ENV coding. In the PC group, no significant amplitude difference in FFR_TFS_ was observed between the quiet and noise-masking conditions, suggesting that since FFR_TFS_ was already degraded in quiet, no further degradation could be observed when the masking noise was introduced. Since the PTA thresholds were negatively correlated with the correlation values for the quiet-to-noise response analysis, the ability of encoding TFS signals in the PC group under the noise condition was weaker than that of the NH group. The results are also consistent with previous reports in SNHL patients. For example, both Buss et al. (2004) and Lorenzi et al. (2006) revealed a decreased ability to use TFS among SNHL patients. Taken together, the results regarding both FFR_ENV_ and FFR_TFS_ in the hearing loss group suggest that patients with impaired audibility cannot adjust the corresponding proportion of the envelope and TFS under noise conditions the way that individuals with NH are able to. The results of this study showed that in listeners with PC, reduced hearing sensitivity could lead to an imbalance of envelope-to-TFS coding under noise conditions, which may be one of the mechanisms underlying speech recognition disorder among listeners with PC. Anderson et al. (2013a) examined the FFR of listeners with PC under noise conditions, and their results were very similar to ours. They compared the amplitude differences between the envelope and TFS representations and found that the differences of the hearing-impaired group were significantly higher than those of the NH group in the noise condition but not significantly different from those of the NH group in the quiet condition, suggesting the presence of an imbalanced envelope-to-TFS representation, especially in noise (Anderson et al., 2013a). The imbalance between the envelope and TFS was also demonstrated in a perceptual study (Fogerty and Humes, 2012).

Note that for native speakers of Mandarin, the envelope information is important for representing lexical tone signals not only in NH listeners under noise conditions (Qi et al., 2017) but also in hearing-impaired listeners (Wang et al., 2011). In this study, the S-R correlation was used to analyze the accuracy of representing the envelope and TFS signals of the sound stimulus. The results showed that the S-R correlations in the PC group were significantly lower than those in the NH group for FFR_ENV_ but not for FFR_TFS_. It is of interest to determine whether elevation of the PTA threshold would also lead to a decrease in the S-R correlation for FFR_ENV_ in speakers of English or other western languages.

### FFR and SIN Performance

In this study, we investigated the correlation between SIN perception thresholds and variables of both FFR_ENV_ and FFR_TFS_. The results showed that lower SIN perception thresholds (better performance) were significantly correlated with higher FFR_ENV_ S-R correlation in the quiet condition, indicating that deficits of subcortical coding of the envelope can affect SIN perception. Lower SIN perception thresholds were also found to correlate with higher H2 (FFR_ENV_) amplitude alterations by noise, i.e., the better the SIN performance is, the greater the H2 amplitude difference between the quiet and noise conditions, suggesting that FFR_ENV_ plays a smaller relative role under noise conditions. This finding further confirms that the change in the envelope-to-TFS encoding ratio under noise conditions is a likely mechanism underlying the speech recognition disorder in noise conditions among listeners with PC. These findings also indicate that FFR may be a useful objective tool to predict SIN perception.

Anderson et al. (2013b) also investigated the correlation between SIN perceptions and speech ABR variables elicited by /da/. Both the self-reported SIN perception and the results of SIN tests were correlated with the speech ABR variables. In their study, a 40-ms /da/ stimulus, which did not include the steady-state part, was used, and variables were mainly from the temporal domain, which were not able to be regarded as a real FFR response. In the present study, we used a much longer stimulus, a 170-ms /da/ stimulus, which had a steady-state part of more than 100 ms, and we primarily analyzed frequency domain variables of the FFR, in addition to response-stimulus correlations. Our results further demonstrated that FFR may be a useful objective tool for predicting SIN perception.

### Influence of Age on the Neural Representation of the Envelope and TFS

Although we did not focus on an analysis of the effect of age on FFR because the participants’ age distribution was continuous, we did analyze the correlations between age and the FFR variables. The results showed that the variability in age across participants was not significantly correlated with the variability in FFRs across participants, even though some previous studies have shown that the age factor affects FFRs (Anderson et al., 2012; Clinard and Tremblay, 2013; Bidelman et al., 2014). It is known that auditory aging is rooted in degenerative alterations in both the peripheral hearing organs (e.g., loss of hair cells) and the central auditory system (e.g., atrophy of the gray and white matter; for a recent review see Ouda et al., 2015). In the present study, older adult participants with PC exhibited a higher PTA threshold, which is related to hair cell dysfunction, than their age-control participants without PC. The results of the present study also demonstrated that the PTA threshold was associated with the noise-induced changes in the TFS component of the FFRs, which reflect the brainstem representations of sound TFS signals in both humans (Wang et al., 2018) and rats (Wang and Li, 2015, 2017, 2018; Luo et al., 2017).

### Limitations

In this study, the FFR tests used the same test signals for the patients with reduced hearing sensitivity and the subjects with NH, i.e., signals with an intensity of 85 dB SPL were used for both groups. However, higher-intensity stimulus signals were used in previous studies of FFRs in SNHL patients. For example, Ananthakrishnan et al. (2016) simultaneously used stimuli with the same sound pressure level and the same sensation level that were used for subjects with NH, while Anderson et al. (2013a) used a stimulus that was modified using the National Acoustics Laboratory-Revised (NAL-R) algorithm according to each individual’s PTA threshold. However, increasing the intensity of the stimulus according to the subject’s hearing threshold does not completely eliminate the impact of hearing sensitivity itself on FFR because even if SNHL patients and individuals with NH are presented signals with the same sensation level, the loudness perceived by the two groups is most likely different due to the presence of recruitment in patients with SNHL. Furthermore, for subjects with a PTA threshold higher than a certain level, the equipment cannot produce stimuli of the same sensation level as that of the other subjects because of its maximum output limitations. In addition, one of the objectives of this study was to examine the effect of hearing sensitivity changes on the FFR, which required simultaneous tests in both noise and quiet conditions. Thus, due to time constraints, we chose to use stimuli with the same intensity and noise to monitor the FFR. In fact, this test condition is truer to the hearing environment in the daily lives of patients with hearing loss, i.e., hearing speech at the same intensity in the same noise background as individuals with NH.

In the present study, we mainly investigated the effect of audibility on subcortical encoding of noise signals in people with PC. Studies have shown that deficits in suprathreshold auditory processing are related to reductions in audibility in people with PC and may occur even without an aging-related elevation in the PTA threshold (Humes et al., 2012; Peelle and Wingfield, 2016). Clearly, further studies evaluating higher-order auditory-processing abilities are needed in the future to clarify the relationship between subcortical encoding and central hearing loss. Furthermore, phonological awareness is an individual’s awareness of the sound structure of words and studies in deaf children have demonstrated alterations of their phonological awareness (Johnson and Goswami, 2010). Thus, future studies investigating the relationship with phonological awareness may also be very informative.

## Summary

In this study, the FFR of subjects with NH and PC was investigated under quiet and noise conditions. In the quiet condition, the amplitudes and S-R correlations of FFR_ENV_ were significantly higher in the NH group than in the PC group. The NH group showed a significantly lower amplitude and S-R correlation of FFR_ENV_ in the noise condition than in the quiet condition, but no similar alterations were observed in the PC group, suggesting that listeners with PC cannot adjust the envelope-to-TFS ratio in noise conditions the same way that individuals with NH are able to. This discrepancy is likely one of the reasons why listeners with PC experience decreased speech recognition under noise conditions. Furthermore, worse SIN performance was observed to have a close relationship with lower S-R correlation in the quiet condition and lower FFR_ENV_ amplitude alteration in the noise condition. These findings further supported that the change in the envelope-to-TFS encoding ratio under noise conditions is a likely mechanism underlying the speech recognition disorder in noise conditions among listeners with PC. In another aspect, these results also indicate that FFR may be a useful objective tool for predicting SIN perception.

## Author Contributions

YS and LL conceived the research. YS, QW, ZG and DN planned the study design. WH and YQ performed the FFR recording. WH and QW analyzed the data. YS, QW and LL wrote the manuscript.

## Conflict of Interest Statement

The authors declare that the research was conducted in the absence of any commercial or financial relationships that could be construed as a potential conflict of interest. The reviewer MM and handling Editor declared their shared affiliation.

## Abbreviations

ABR: auditory brainstem response
ENV: envelope
FFR: frequency following response
NH: normal hearing
PC: presbycusis
PTA: pure tone audiometry
SIN: speech-in-noise
SNHL: sensorineural hearing loss
SNR: signal-to-noise ratio
TFS: temporal fine structure.
